# Hemiballismus–hemichorea syndrome in an acute rehabilitation setting: two case reports 

**DOI:** 10.1186/s13256-022-03577-3

**Published:** 2022-10-31

**Authors:** Luke Tsai, Benjamin Shekhtman, Ryan Stenquist, Ashley Schneider, Meilani Mapa, Edwin Amirianfar, David R. Gater

**Affiliations:** 1 Department of Physical Medicine and Rehabilitation, University of Toledo College of Medicine and Life Sciences, 3000 Arlington Ave, Toledo, OH 43614 USA; 2 Department of Physical Medicine and Rehabilitation, University of Miami Miller School of Medicine, Miami, Florida USA; 3 The Miami Project to Cure Paralysis, University of Miami Miller School of Medicine, Miami, Florida USA

**Keywords:** Basal ganglia, Dyskinesias, Hyperglycemia, Rehabilitation, Case report

## Abstract

**Background:**

We report two similar cases of patients diagnosed with hemiballismus–hemichorea syndrome secondary to uncontrolled hyperglycemia. Both patients were treated at an inpatient rehabilitation center and made a significant recovery in both function and activities of daily living. Although hemiballismus–hemichorea syndrome has known pharmacological treatments, little has been reported on the effectiveness of acute rehabilitation on managing and treating this syndrome.

**Case presentation:**

The first case involves a 44-year-old Hispanic male with past medical history of type 2 diabetes mellitus, hypertension, anxiety, and depression who presented with continuous, uncontrollable, unilateral movements 1 month following a hospital admission for hyperglycemia. Magnetic resonance imaging findings showed lesions in the basal ganglia, confirming the diagnosis of hemiballismus–hemichorea syndrome. The patient was started on antipsychotic medications and antihyperglycemic medications controlling glucose levels, but continued to have hemiballismus symptoms. The second case involves a 78-year-old Haitian female with past medical history of type 2 diabetes mellitus and hypertension who presented with weakness and continuous, involuntary movements in her upper and lower extremities 1 month following a hospital admission for hyperglycemia and encephalopathy. Magnetic resonance imaging findings were positive for bilateral lesions in the basal ganglia, establishing a diagnosis of hemiballismus–hemichorea syndrome. After a 2-week stay at an acute rehabilitation center, both patients made a significant recovery in function and activities of daily living.

**Conclusion:**

The aim in presenting these cases is to elucidate the etiology and progression of a rare disease process known as hemiballismus–hemichorea syndrome and to provide evidence for the potential positive impact of acute rehabilitation on patients with unresolved hemiballismus–hemichorea following an episode of hyperglycemia.

## Background

Hemiballismus–hemichorea (HBHC) syndrome can be defined as a hyperkinetic movement disorder characterized by involuntary, irregular, continuous, unilateral movements of the body resulting from a contralateral basal ganglia lesion. The most common etiologies include ischemic stroke, hemorrhagic stroke, and autoimmune conditions [1]. Although rare, hyperglycemia is another potential cause of HBHC syndrome [2], also known as chorea–hyperglycemia–basal ganglia syndrome in this context. Although the exact pathophysiology is unknown, one proposed mechanism suggests that hyperglycemia increases the blood viscosity and disrupts the blood–brain barrier, consequently leading to a temporary ischemia of the neurons in the basal ganglia, one of the most susceptible areas of the brain [1]. Another theory suggests that decreased perfusion and ischemia leads to a shift towards anaerobic metabolism and consequently increased metabolism of γ-aminobutyric acid (GABA), the main inhibitory neurotransmitter of the basal ganglia [3].

Diagnosis is typically based on the presence of the triad of chorea, hyperglycemia, and striatal abnormalities on neuroimaging. Radiographic findings include contralateral striatal hyperintensity on T1-weighted images and/or hyperdensity on noncontrast computed tomography (CT) scans [4]. For management of HBHC syndrome, it is essential to treat the underlying cause of hyperglycemia. Normalization of blood glucose typically resolves chorea symptoms, however there are known cases of chorea continuing for weeks even after correction of hyperglycemia [5, 6]. For addressing chorea symptoms, dopamine antagonists such as typical and atypical antipsychotics along with vesicular monoamine transporter (VMAT) inhibitors such as tetrabenazine can be used as first-line treatments [7]. Notably, the presence of chronic medical illness and use of steroids may worsen the prognosis of this condition [8].

This study reports two similar cases of patients diagnosed with HBHC syndrome secondary to uncontrolled hyperglycemia. Both patients were treated at an inpatient rehabilitation center and were able to make significant recovery of function and activities of daily living (ADLs). Though HBHC syndrome has known pharmacological treatments, little has been reported on the effectiveness of acute rehabilitation on managing and treating HBHC syndrome. These cases aim to elucidate the progression of HBHC syndrome in an acute rehabilitation setting. Written patient consent was obtained from both patients for the publication of this case report.

## Case presentation

### Case 1

A 44-year-old Hispanic male with past medical history of type 2 diabetes mellitus, hypertension, anxiety, and depression and new diagnosis of HBHC syndrome based on correlating clinical and magnetic resonance imaging (MRI) findings was admitted to an acute rehabilitation hospital for management of symptoms. On admission, initial examination revealed continuous, uncontrolled spasms of the right arm and leg. The patient relied on maximum assistance or was dependent on 2+ helpers for all aspects of self-care. For functional mobility, he required a supervisor/touch assistance for bed mobility, and partial to maximum assistance for transfers.

The patient initially presented to the hospital 6 weeks prior for elevated blood glucose levels in the range of 400–500. The patient did not require intubation and did not have any altered mental status during the hospital course. At the time of discharge, his blood glucose was controlled but his wife stated that, upon returning home, they remained elevated in the 400–500 range. Notably, with the patient’s history of anxiety and depression, he had been started on quetiapine 50 mg nightly earlier in the year, which was subsequently changed to olanzapine 7.5 mg prior to this admission.

The patient had another hospital admission 2 weeks prior with 2-day history of involuntary movements in his right upper and lower extremities. He was evaluated with brain CT, MRI, and EEG and was discharged on haloperidol 5 mg twice daily for his hemiballismus symptoms. EEG was negative for epileptic waveforms, while CT and MRI findings were consistent with changes of nonketotic hyperglycemia and clinical correlation for HBHC syndrome. During this admission, laboratory values were positive for mild leukocytosis, blood glucose level of 125 mmol/L, and an A1C of 14.6%. Blood workup for secondary causes of hemichorea was unremarkable. The patient was admitted under the neurology service and was started on clonazepam 2 mg TID and quetiapine 25 mg TID for his hemiballismus symptoms, though with little improvement. Endocrinology was consulted for control of his blood glucose, and he was started on Lantus and sliding scale insulin therapy before discharge to the acute rehabilitation hospital.

**Fig. 1:**
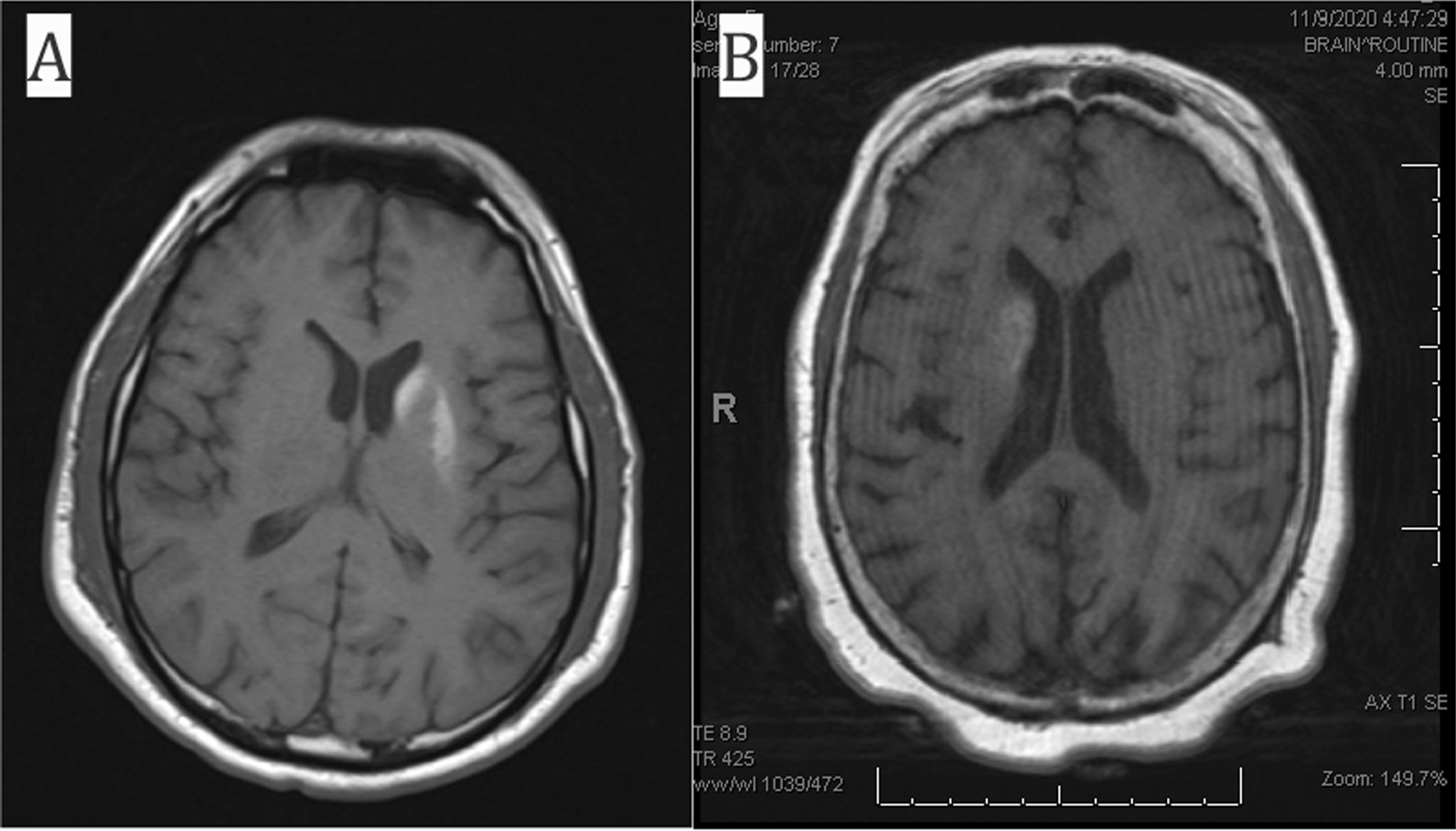
Brain MRI T1-weighted axial images representing basal ganglia lesions secondary to hyperglycemia in HBHC syndrome patients. **A** Diffuse hyperintensity of the left caudate and lentiform nucleus seen in a 44-year-old Hispanic male from case 1. **B** Diffuse hyperintensity of the right caudate nucleus seen in a 78-year-old Haitian female from case 2

During his 2-week-long admission at the rehabilitation hospital, the patient received daily focused therapy while proper blood glucose control was maintained. He was evaluated by a neurologist, who planned to place him on tetrabenazine which would be started 2–3 weeks after insurance approval. At the time of his discharge, significant improvements in all aspects of self-care, bed mobility, and transfers were appreciated. The patient received a follow-up MRI scan with and without contrast 3 weeks after discharge, which showed continued signal abnormality in the left lentiform nucleus with development of signal in the left caudate nucleus (Fig. 1A). Palliative care was consulted, and at a subsequent visit, symptoms continued to improve with right-sided leg movements drastically decreased. Speech was also noted to be clearer with the patient able to express himself more fluently. The patient was walking with the use of his walker and able to perform his own ADLs. Physical therapy and occupational therapy were completed at home, and a plan was made to continue outpatient physical therapy. Tetrabenazine was not initiated due to improvement in symptoms. Patient continues to be on insulin with well-controlled blood glucose levels, with his latest A1c at 6.1%, significantly decreased from 14.6% at initial presentation to the hospital.

### Case 2

A 78-year-old Haitian female with type 2 diabetes mellitus and hypertension was admitted to the inpatient rehabilitation unit for chorea secondary to new bilateral basal ganglia T1 hyperintensities on MRI, particularly in the right caudate nucleus, favored to be hyperglycemia related. She was initially admitted to a level 1 tertiary center with 3-day history of involuntary upper and lower extremity movements that progressively worsened with generalized weakness compromising her ability to grasp utensils and ambulate. There were no associated symptoms of headache, fever, weight or appetite change, loss of consciousness, blurred or double vision, dysphagia, or numbness. Furthermore, there were no recent medication changes. One month prior, she had been admitted for encephalopathy, with hyperglycemia greater than 700 and MRI imaging with diffuse low T2 signal in bilateral basal ganglia secondary to hyperglycemia (Fig. 1B).

Aripiprazole 5 mg twice a day was started and continued during her rehabilitation admission. Glucose levels were tightly controlled and maintained under 200 except for three isolated episodes over her 14-day rehabilitation stay. She was admitted to inpatient rehabilitation to work with physical therapy, occupational therapy, and speech therapy to work on her deficits (Table 1). Prior to this, she was independent with ADLs, transfers, and mobility. Both of her physical and occupational therapy evaluations noted choreiform movements while sitting at the edge of the bed and at rest in the bilateral upper extremities that decreased with volitional movement; dystonia was present in static standing in the right lower extremity. Speech therapy noted a mild to moderate dysarthria with reduced speech intelligibility. Choreiform movements, dystonia, cognitive deficits, and dysarthria with reduced speech intelligibility were her main barriers in therapy.

**Table 1:** Improved functional independence measures from admission to discharge for a 78-year-old Haitian female patient from case 2

	Admission assessment	Discharge assessment
Physical therapy		
Bed mobility	Independent	Independent
Transfers	Minimal assistance	Supervision
Gait	Minimal assistance (150 feet)	Supervision (150 feet)
Steps	Minimal assistance (12 steps)	Supervision (12 steps)
Wheelchair mobility	Maximal assistance (150 feet)	Total assistance (150 feet)
Occupational therapy		
Eating	Supervision	Independent
Oral hygiene	Supervision	Independent
Upper body dressing	Supervision	Independent
Lower body dressing	Minimal assistance	Supervision
Bathing	Minimal assistance	Supervision
Shower/toilet transfers	Minimal assistance	Supervision
Toileting	Moderate assistance	Supervision
Speech therapy		
Comprehension	Minimal assistance	Standby
Expression	Minimal assistance	Standby
Social interaction	Moderate assistance	Standby
Memory	Moderate assistance	Minimal assistance
Problem solving	Maximal assistance	Moderate assistance

Physical therapy techniques used for chorea and dystonia included volitional movements (that is, grabbing or holding a cup of water); cognitive distraction, prolonged exhalation, meditation; ambulation and weight bearing; balance in parallel bars; visual feedback using mirrors; gait training with and without an assistive device. Occupational therapy techniques for chorea and dystonia included energy conservation techniques using figure-4 technique; endurance exercises including ergometer; deep vibration with noted reduction in chorea movements; mass practice task consisting of grasping and transferring rings from right to left with hand over hand; functional grasp tasks; visual feedback to increase standing unsupported tolerance in preparation for ADLs participation; Sander box with 3-pound dumbbell using mirror as visual feedback; Nu Step reciprocal stepper for gross motor coordination and activity endurance. Speech therapy techniques for cognitive deficits included targeted cognition and dysarthria as well as compensatory techniques.

Nine months after discharge from acute inpatient rehabilitation, she indicated that she continues to improve, not only in function but with her tremor as well. In addition to the therapies that she received to help her return home and increase independence, she stated that inpatient rehabilitation had helped formulate a home exercise program that she continues to do on a regular basis. She also reported that she is no longer using a walker and is ambulating independently. She stated that all aspects of her therapy, including gait training, were equally beneficial for her recovery. She was pleased with her rehabilitation course and ecstatic about her outcome.

## Discussion

HBHC syndrome is a hyperkinetic disorder caused by damage to basal ganglia structures, which can be a result of uncontrolled hyperglycemia. HBHC symptoms typically resolve over the course of weeks to months after proper glycemic control is obtained but can be prolonged or even permanent in some cases [2]. While medications such as typical and atypical antipsychotics may help control symptoms, little has been reported about the efficacy of acute rehabilitation on patient functionality and quality of life.

These cases demonstrate the effectiveness of acute rehabilitation as it is uniquely equipped to address many of the initial safety and functional concerns that HBHC syndrome creates. Through patient/family education and directed therapy, concerns such as self-harm from uncontrolled choreiform movements, safety with ambulation, practicalities of performing ADLs, and establishing a safe, patient-friendly living environment can all be adequately assessed and evaluated. Furthermore, a comparison of the admission and discharge assessments for both patients illustrates significant improvements in function and ADLs after inpatient rehabilitation (Table 1). One limitation of this study is the multifactorial nature of the intervention as both patients had pharmacological treatments in addition to acute rehabilitation, which includes various types of therapies. Further research would be required to isolate the specific aspects of acute rehabilitation that most significantly impact the recovery stage in HBHC syndrome.

## Conclusion

The significant improvements made by both patients in self-care and functionality provide evidence for the positive potential impact that acute rehabilitation can have in patients with continued unresolved HBHC syndrome after an acute uncompensated hyperglycemic episode. Though HBHC is a rare disease process, it is important to be aware of its presentation and to emphasize glucose control, management of chorea symptoms, patient education, and directed therapy for optimal treatment.

## Data Availability

Not applicable.

## Abbreviations

HBHC: Hemiballismus–hemichorea
ADLs: Activities of daily living
GABA: γ-Aminobutyric acid
CT: Computed tomography
VMAT: Vesicular monoamine transporter
